# Child Maltreatment During School and Childcare Closure Due to the COVID-19 Pandemic

**DOI:** 10.1177/10775595211064885

**Published:** 2022-02-01

**Authors:** Samantha Vermeulen, Lenneke R. A. Alink, Sheila R. van Berkel

**Affiliations:** 1 Institute of Education and Child Studies, 100575 Leiden University, Leiden, Netherlands

**Keywords:** child maltreatment, prevalence, lockdown, COVID-19 pandemic, socio-demographic risk factors

## Abstract

The aim of the present study was to examine child maltreatment prevalence rates during the first COVID-19 related national closure of schools and childcare settings (the lockdown) in the Netherlands. Based on reports of childcare professionals and primary and secondary school teachers (*N* = 444) the prevalence of child maltreatment during the 3 months of this first lockdown was estimated at almost 40,000 children, or 14 per 1,000 children. The prevalence of emotional neglect was found to be three times higher during the lockdown compared to a period without lockdown. This significant difference was reflected in overall emotional neglect as well as for two main subtypes of emotional neglect: educational neglect and witnessing domestic violence. No significant differences were found for other types of child maltreatment. Most of the reported cases of maltreatment were already problematic before the lockdown and became worse during the lockdown. The results of this study indicate that the closure of schools and childcare settings may have enormous negative consequences for vulnerable children.

The SARS-CoV-2 virus, also known as the Coronavirus, has spread rapidly across the globe since the end of 2019. In March 2020, the World Health Organization (WHO, 2020) officially announced the COVID-19 outbreak a pandemic. As in many countries, also in the Netherlands several national policy measures have been enforced to slow down the spread of the virus. On March 15, 2020, the so-called “*intelligent lockdown*” was implemented by the Dutch Government to flatten the curve. During this lockdown people were requested to work from home as much as possible, social gatherings were prohibited, contact with people outside of the household was discouraged, people were allowed to be outside with a maximum of three people while keeping 1.5 m of distance, pubs and restaurants were closed, and schools and child care settings were closed (Rijksoverheid, 2020a). Although the lockdown seemed to have been effective to flatten the curve, as also supported by results from studies on lockdowns in other countries (for a review, see Viner et al., 2020), at the same time there are concerns that the measures might have led to an increase in child maltreatment and domestic violence in some of the families (Abramson, 2020; UNICEF, 2020). The aim of the current study is, therefore, to estimate the prevalence of child maltreatment during the lockdown and to compare this estimate to a period without lockdown, based on data from the Netherlands’ Prevalence study of Maltreatment of children and youth from 2017 (NPM-2017; Van Berkel et al., 2020).

The lockdown drastically changed the daily lives of many families, which may have increased the risk on child maltreatment. First, the combination of working from home and simultaneously supporting the children in their online education might have increased the levels of stress for parents. Results from several online surveys indeed show that parents experienced higher levels of stress during the lockdown due to the highly demanding and challenging situation (Landelijk Ouderpanel, 2020; Wessels, 2020). Experiencing high levels of stress might make it harder to stay positive and keep warm relations with each other. Indeed, parents and adolescents reported a decrease in positive interactions during the lockdown, and this effect appeared to be stronger in families with higher levels of stress (Donker et al., 2020). Moreover, high levels of parenting stress are an important risk factor for child maltreatment (Stith et al., 2009), suggesting that increased levels of stress might in turn have increased the risk for child maltreatment during the lockdown.

Another reason why the lockdown could have led to unsafe home situations could be the fact that family members were forced to spend more time with each other and had less personal space and private time, which might have increased frustrations and irritabilities between family members (Bröer et al., 2021). Frustrations and irritabilities could result in feelings of anger and aggression (Berkowitz, 2012), which could in turn be reflected in overreactive parenting and heighten the risk for the use of more abusive behaviors (Rodriguez et al., 2015). This might be particularly true for families with pre-existing psychological problems or with a history of child maltreatment or domestic violence. Whereas children of parents with psychological problems or partners of individuals with psychological problems normally can escape the situation at home by attending school, working outside the home or engaging in social interactions, they had to deal with the problems of their parent or partner 24/7 during the lockdown. This could have increased levels of stress and negative feelings during the lockdown, which may enhance the risk on child maltreatment and domestic violence (Achterberg et al., 2021). The decreased options to access services and social support systems might even have exacerbated these problematic situations. Also, in families with a history of domestic violence or child maltreatment, the lockdown might have reduced possibilities for victims to ask for help or to escape from their perpetrator, and so this might have increased the likelihood of exposure to domestic violence or maltreatment during the lockdown.

Moreover, unemployment and financial stress might have been other consequences of the lockdown that may increase the risk on child maltreatment. Unemployment rates increased fast during the lockdown in the Netherlands, from 2.9% in February to 4.3% in June 2020 (Statistics Netherlands Centraal Bureau voor de Statistiek [CBS], 2020a). From previous research, it is known that unemployment and parenting stress are related to an increased risk for child maltreatment (Stith et al., 2009; Van Berkel et al., 2020). The increased levels of stress, the pressure on all members of the family, and the increased unemployment rates could have resulted in an increase in prevalence rates of child maltreatment during this period. Also, the lack of social control due to the social restrictions, including less face-to-face contact with youth assistance agencies, could have contributed to this.

Results from studies and numbers of official reports on child maltreatment and domestic violence from all over the world seem to be inconsistent. Direct comparisons between different countries are hard to make, since lockdown measures differed from country to country. Still, in general, most news reports and studies based on official reports seem to indicate no change at all or even a decrease in child maltreatment and domestic violence during the lockdown (ABC News, 2020; Baron et al., 2020; CBS, 2020b; Đapić et al., 2020; Li & Schwartzapfel, 2020; Stone et al., 2020) while results from most studies based on self-report indicate an increase (Het Kinderrechten Commissariaat et al., 2020; Sayfa Çalişmalari, 2020; Vandeviver et al., 2020). In addition, findings from online hotlines and chat services also signaled an increase in phone calls and chats from people with questions regarding unsafe home situations (De Kindertelefoon, 2020a, 2020b; Van Bemmel et al., 2020). Noticeable to this increase is the fact that most of these unsafe situations seemed to have existed a long time before the lockdown already, suggesting that situations might have gotten worse during the lockdown. Taken all of this together, we hypothesize that there has been an increase of child maltreatment during the lockdown which has not sufficiently been reported by professionals and the social network to child protection organizations.

Previous research has shown that a low educational level, unemployment, immigrant status, single parenthood, stepfamilies, large families, and young age of the child increase the risk for child maltreatment (Assink et al., 2016; Belsky, 1980; Stith et al., 2009; Van Berkel et al., 2020). The lockdown might have had a different impact on families with different characteristics, as outlined above. Getting a better understanding of factors that increased the risk for child maltreatment during the lockdown could help policy makers to provide more support for vulnerable families during future lockdowns.

Given the negative short and long-term consequences of child maltreatment on the mental and physical health from childhood till adulthood (Norman et al., 2012), and given the long-term nature of the pandemic, examining whether child maltreatment has increased during the lockdown compared to a period without lockdown is essential. Knowledge about child maltreatment during this first lockdown might help to inform policy in future lockdowns. The present study, therefore, investigates the prevalence of child maltreatment during the first COVID-19 lockdown in the Netherlands based on reports from informants working in childcare settings, and primary and secondary school teachers (based on the method used in the National Prevalence of Maltreatment study 2017 [NPM-2017], Van Berkel et al., 2020). The main goal of the current study was to estimate the number of children who were victim of child maltreatment during the first lockdown (March 16, 2020–June 16, 2020) in the Netherlands and compare it with the prevalence of a period without a lockdown. Additionally, this study aims to explore what socio-demographic child and family factors increased the risk for maltreatment during the lockdown.

## Method

### Participants

Professionals working in childcare settings (home-and center-based childcare and kindergartens), and teachers working in primary and secondary education participated in this study. The sampling procedure is based on previous NPM-studies (NPM-2005: Euser et al., 2010; NPM-2010: Euser et al., 2013; NPM-2017: Van Berkel et al., 2020). As in the previous NPM-studies, the Netherlands was divided into five zones with approximately equal numbers of children living in each zone. Then the total number of organizations and professionals per zone was determined in such a way that the professionals in each of the zones covered approximately equal numbers of children to obtain a geographically representative sample. A total of 630 childcare organizations, 500 primary schools and 225 secondary education schools were invited to participate in the study. They were contacted to participate in the study by email. Directors of childcare centers were asked to send the survey randomly to one childcare worker per group within their organization. School principals were asked to send the survey to all teachers within primary schools (if there were multiple teachers in one class, only one of them was asked to participate) and to all teachers with their own mentor class in high schools.

The invitations for this study were sent in the week after schools completely reopened again. All schools and childcare centers had been completely closed for a period of 2 months. After this period childcare centers were allowed to completely reopen again, while primary schools could only reopen partially (children were allowed to go to school for 50% of the time), and secondary schools had to remain closed for one more month. In total, schools and childcare centers have been (partially) closed for a period of 3 months, from March 16^th^ till June 15^th^ (Rijksoverheid, 2020b). The period right after the lockdown was extremely busy, especially for teachers, which led to a high non-response rate in this occupational branch. For this reason, we decided to invite a new sample of professionals for participation in our study in the period directly after the summer break (September 2020).

In total 444 professionals participated in the study; 273 in the first phase of the study (before the summer break) and 171 in the second phase of the study (Table 1). Since participation was completely anonymous, no information is available about the number of organizations that participated. Based on postal codes, we know that the 444 professionals were working in organizations in 309 different postal code areas, equally divided over the five zones of the Netherlands (18.1%, 20.4%, 21.1%, 20.4%, and 20.0% for zone 1 to 5, respectively). The professionals and teachers reported on a total of 2,254 children in center-based childcare, 1,153 children in home-based childcare, 1,629 children in primary schools and 1881 children in secondary schools (Table 1).

**Table 1.** Total Number of Participating Professionals, Sample of Observed Children, and Total Number of Children per Occupational Branch.

Occupational branch	Professionals			Observed children^a^			Total Dutch population^a^
	Phase 1	Phase 2	Total	Phase 1	Phase 2	Total	
Center-based childcare^b^	80	25	105	1,722	533	2,255	469,530
Home-based childcare	147	44	191	872	281	1,153	145,430
Primary schools	17	49	66	418	1,211	1,629	1,396,575
Secondary schools	29	53	82	675	1,206	1,881	950,447
Total	273	171	444				

^a^The samples of observed children and the total Dutch population cannot be summed to a total per phase, since children in home-based childcare and primary schools can be observed by professionals from both occupational branches.

^b^Center-based childcare also includes kindergartens.

### Procedure

The procedure and design of this study were approved by the Ethics Committee of Education and Child Studies (ECPW-2020/277). After a digital informed consent was obtained from the participants, they filled out a short survey and a standardized online registration form based on the form used in the previous NPM-studies (Euser et al., 2010, 2013; Van Berkel et al., 2020). Professionals were asked to participate in the study regardless of whether they had suspicions of child maltreatment during the 3-month research period (March 16, 2020–June 16, 2020). In the first survey, professionals were asked to indicate the number of children in their professional population. In the second phase, participants were also asked for how many children of their professional population they suspected maltreatment. In addition, for each case of suspected child maltreatment participants anonymously filled out the digital registration form which consisted of questions about the child, the home situation, parents or other important caregivers, the suspected child maltreatment, domestic violence, and whether professionals thought the situation might have changed during the lockdown.

Professionals reported (suspicions of) child maltreatment for a total of 59 children. One of those reports was excluded from analysis because the description of the situation was not considered to be maltreatment based on the definitions used in this study (see below). Further, we checked whether different participants reported about the same child, but no duplicate cases were found in the current study. This resulted in the inclusion of a total of 58 cases of suspected child maltreatment of children living in 56 families: seven children in the first phase of the study and 51 children from 49 different families in the second phase of the study.

### Coding of Maltreatment

The descriptions of the suspected cases of child maltreatment were independently coded by three trained coders (two of them also coded cases in the NPM-2017) to decide whether the cases were considered child maltreatment based on the definitions used in the previous NPM-studies (Euser et al., 2010, 2013; Van Berkel et al., 2020) and in the National Incidence Studies in the USA (NIS; Sedlak et al., 2010). If the information provided in the report did not meet the definitions of maltreatment, the report was not included in the analysis. Cases of child maltreatment were classified into one or more of the six maltreatment types according to the definitions: (1) sexual abuse, (2) physical abuse, (3) emotional abuse, (4) physical neglect, (5) emotional/educational neglect, and (6) other abuse or neglect. All cases were double coded: the new coder coded all cases and each of the expert coders (who had been involved in coding in previous NPM-studies) coded half of the cases. The percentages of agreement between the two pairs of coders concerning the classification of the maltreatment into one or more types were 93.3% and 98.3%. In case of disagreement, the case was discussed with the third coder to reach consensus.

### Statistical Procedures

The prevalence rates of child maltreatment during the lockdown in the Netherlands were estimated for both phases separately and for both phases combined. The prevalence estimates for each occupational branch and each type of child maltreatment were computed using the following formula

$$\text{X }=\;\frac{\text{C}}{\text{TOT}s}\;\text{ * TOT}\mathrm{pop}$$

In this formula, x represents the estimation of the number of maltreated children, C is the number of reported cases during the lockdown, TOT*s* is the total number of (potentially) observed children by the professionals from an occupational branch, and TOT*pop* represents the total number of Dutch children belonging to an occupational branch of professionals (Euser et al., 2010, 2013; Van Berkel et al., 2020).

The number of observed children and the total population of children for each occupational branch is presented in Table 1. To determine the number of (potentially) observed children by the professionals from an occupational branch, the professionals reported the number of unique children per week they had contact with just before the lockdown. The total number of Dutch children belonging to an occupational branch of professionals was determined using the most recent data available through public data of Statistics Netherlands (StatLine). Although the large majority of children in the Netherlands are seen by one of the professionals of these three occupational branches we included in the study, not all children are included in the populations of these professionals (e.g., preschoolers who do not use any form of childcare, children who attend special education or adolescents under the age of 18 who finished secondary education already or dropped out of school). Hence, estimates of the number of children who have been maltreated during the lockdown in the Netherlands in the current study are based on all children under the age of 18 who make use of either some form of childcare, or primary education, or secondary education (Table 1).

### Comparison With a Period Without Lockdown (NPM-2017)

To compare the relative prevalence estimates (the estimate per 1000 children) from this period with the prevalence estimates from a 3-month period without lockdown, data from the NPM-2017 was used, since the design from the present study was almost similar to the design of the NPM-2017 (i.e., similar recruitment procedure, registration form, duration of research period, and coding procedure). The data from the NPM-2017, based on reports from occupational branches included in the present study only (i.e., only reports from of childcare professionals and primary and secondary school teachers), were reanalyzed in the same way the data of the present study was analyzed to make reliable comparisons. This means that the data collected in 2017 were not extrapolated to an annual prevalence estimate (which was done for the NPM-2017), but instead the formula used in the present study was used for computation of the prevalence estimates for both 3-month periods: The period in 2017 and the period during the lockdown. In addition, to test whether results were similar if we control for seasonality (both studies took place during different seasons) we calculated a seasonal factor for both periods based on the Safe at Home (CPS) data which was collected in earlier NPM-studies (Euser et al., 2013; Van Berkel et al., 2020). The calculated seasonal factors were 1.11 and 0.95 for the NPM-2017 and lockdown period, respectively. As sensitivity analyses, we calculated prevalence rates accounting for these seasonal factors.

The characteristics of the areas served in the 2017 study and the current study were compared based on postal code areas of the participating organizations to determine to what extent the samples and areas served were comparable and representative for the population. The difference between the average degree of urbanity in the areas served in the current study (*M* = 3.27, *SD* = 1.40) compared to the NPM-2017 (*M* = 3.11, *SD* = 1.31) was not significant (*t*(904.54) = 1.77, *p* = .078). In addition, a significant difference was found in the average percentage of households using social security benefits between the two samples, *t*(940) = 6.52, *p* < .001, with the percentage being lower in the present study, *M* = 16.53%, *SD* = 6.08%, compared to 2017, *M* = 19.28%, *SD* = 6.76%.

To determine whether the prevalence estimates significantly differ from each other, 84% confidence intervals were calculated for each prevalence estimate. The use of 84% confidence intervals for significance testing leads to a probability of overlap of approximately 5% (*p* < .05; Goldstein & Healy, 1995; Julious, 2004; Payton et al., 2003). Estimates are considered to differ significantly from each other when there is no overlap between the confidence intervals.

### Risk Factors

The distribution of the child and family characteristics within the present study was compared to the distribution of the characteristics in families with children in the general Dutch population. To do so, the same non-public microdata from Statistics Netherlands (CBS) that was used in the NPM-2017 was used for the present study. Based on the public data from 2017 to 2020 available on StatLine (in which data is represented for the whole population and not specifically for families), it could be argued that the distribution of risk factors in families in the population in 2020 hardly differs from the distribution of risk factors in 2017.

Risk ratios (RR) were computed the same way as in the NPM-2017 (Van Berkel et al., 2020); the proportion of maltreated children within the group exposed to the risk factor was divided by the proportion of maltreated children within the group not exposed to that risk factor. Most of the characteristics were known by most of the participants, but there were some family characteristics with missing values: information was missing on parental education for 62.5% of the families, on parental unemployment for 21.4% of the families, and on immigration status for 11.7% of the families. For the computation of the RR, the missing data was handled by using pairwise deletion, all cases with available data were analyzed. Also, 95% confidence intervals were computed around the RR to represent the precision of each estimate (Rothman, 2002). The characteristics could be considered significant risk factors for maltreatment when the whole confidence interval does not include and is higher than the value of 1.

## Results

### Prevalence Estimates

Since the study consisted of two parts, prevalence estimates were computed for each phase separately and for the two phases combined. Based on reports of maltreatment from professionals in the first phase of the study it was estimated that 10,323 (95% CI: 0.0–24,393) children experienced at least one form of child maltreatment during the lockdown in the Netherlands. Reports in the second phase of the study resulted in a prevalence estimate of 52,518 (95% CI: 29,815–75,221). The prevalence estimates and 84% confidence intervals for each of the occupational branches and the total prevalence estimate are presented in Table 2. Results presented in this table indicate that, compared to the prevalence estimate of the NPM-2017 (estimate: 14,268; 84% CI: 8,435–20,101), reflecting a 3-month period before the pandemic, the total prevalence estimate from the first phase was not significantly different, while the prevalence estimate based on the second phase was significantly higher than the prevalence estimate in the NPM-2017.

**Table 2.** Prevalence Estimates and 84% Confidence Intervals (CI) per Phase per Occupational Branch.

Occupational Branch	Phase 1		Phase 2		Phases Combined		NPM-2017	
	Estimate	84% CI	Estimate	84% CI	Estimate	84% CI	Estimate	84% CI
Center-based childcare	818	153–1,483	2,643	497–4,788	1,249	531–1,967	1,009	398–1,619
Home-based childcare	0	––	2,067	620–3,514	504	149–859	1,540	460–2,620
Primary education	6,689	36–13,343	41,502	31,895–51,108	32,578	25,214–39,942	5,173	3,687–6,659
Secondary education	2,816	13–5,619	6,306	3,173–9,439	5,053	2,806–7,300	6,546	3,890–9,202
Total	10,323	201–20,444	52,518	36,185–68,850	39,385	28,701–50,070	14,268	8,435–20,101

Since the response rate per occupational branch differed between the two phases (see Table 1), with the response rate for child care organizations being higher in the first phase and the response rate for educational organizations being higher in the second phase, the prevalence estimate based on the data from both phases combined was considered more reliable than the estimates per phase. By combining the two samples, non-response bias in each of the separate phases might be reduced and estimates will be based on a larger total sample, resulting in more reliable estimates. The combination of the data of both phases resulted in a prevalence estimate of almost 40,000 children, (estimate: 39,385; 95% CI: 24,533–54,237). This indicates that during the first lockdown of 3 months about 14 per 1000 children were victim of child maltreatment in the Netherlands (95% CI: 8–19 per 1,000 children). This estimate was significantly higher than the prevalence estimate of the NPM-2017, reflecting a 3-month period before the pandemic (estimate: 14,268; 84% CI: 8,435–20,101). In addition, when accounting for potential seasonal effects, the prevalence estimate of the lockdown (estimate: 37,416; 84% CI: 26,997–47,834) was also significantly higher than the prevalence estimate of the NPM-2017 (estimate: 15,802; 84% CI: 9,665–21,938).

### Prevalence of Different Types of Maltreatment

**Figure 1.**
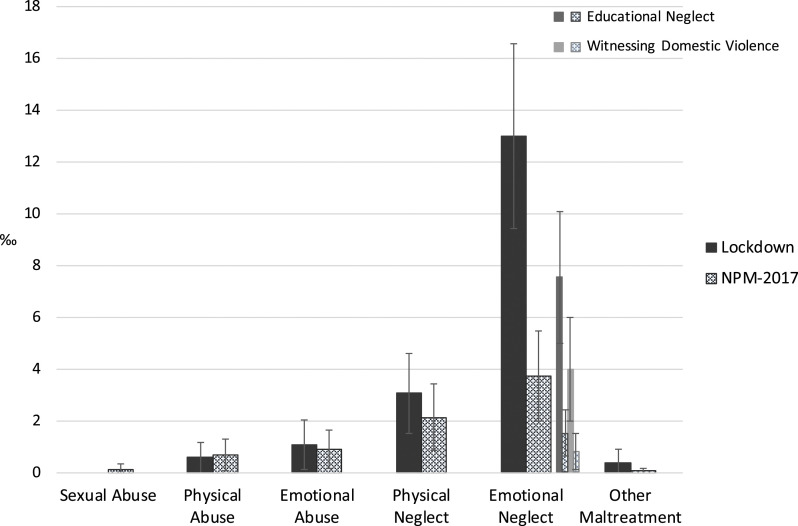
Prevalence estimates of the different types of child maltreatment during the lockdown and in 2017 (including 84% confidence intervals), based on the same occupational branches.

Additionally, prevalence estimates for each type of child maltreatment were computed (see Figure 1). Emotional neglect was the most prevalent type of child maltreatment (37,688; 95% CI: 23,280–52,096). Within this type of maltreatment, we computed separate prevalence estimates for two relatively common subtypes: educational neglect (21,893; 95% CI: 11,643–32,143) and witnessing domestic violence (11,533; 95% CI: 3,429–19,637). The second most prevalent type of child maltreatment was physical neglect (8,883; 95% CI: 2,697–15,069), followed by emotional abuse (3,077; 95% CI: 0–6,975), physical abuse (1,715; CI: 0–4,090), and lastly other maltreatment (1,066; 95% CI: 0–3,153). Sexual abuse was not reported in the present study. The inclusion of the value of 0 in the confidence intervals of emotional abuse, physical abuse, and other maltreatment raises concerns about the reliability of these estimates, since it cannot be ruled out that the actual estimate is zero. Furthermore for 32.8% of the children two forms of child maltreatment were reported. All children who were physically abused or emotionally abused also experienced some form of neglect.

Comparisons with the NPM-2017 estimates showed significantly higher estimates for emotional neglect during the lockdown (Figure 1). This difference was reflected in overall emotional neglect as well as in two main subtypes of emotional neglect: prevalence rates of overall emotional neglect as well as educational neglect and witnessing domestic violence were higher during the lockdown compared to a period without lockdown. For the other types of maltreatment, the comparison showed no significant differences in prevalence rates.

### Unreported Suspicions of Maltreatment

During the second phase of the study, participants were asked to indicate for how many children they suspected child maltreatment during the lockdown, before they were redirected to the specific maltreatment registration form. While they reported suspicions of maltreatment for 91 children, they only filled out a registration form for 40 of those (44.0%). This implies that the prevalence rate may have been higher than the prevalence presented based on complete registrations. When all suspicions of maltreatment were included in the computation of the prevalence estimate of child maltreatment this resulted in a prevalence estimate of 92,989 (84% CI: 70,863–115,115). This is significantly higher than the prevalence estimate of maltreatment based on the coded cases of maltreatment during the second phase only (estimate: 52,518; 84% CI: 36,185–68,850). This prevalence estimate should be interpreted with caution, since these suspicions of child maltreated were not coded and we cannot be sure whether all of these cases were indeed forms of child maltreatment and we did not collect data on maltreatment suspicions in the first phase of the study. However, this estimate does demonstrate that the prevalence rates based on the coded reports of child maltreatment are rather an underestimation than an overestimation.

### Changes in (Unsafe) Home Situations During the Lockdown

For each reported case of child maltreatment participants were asked to indicate whether the suspected maltreatment had changed due to the lockdown. In only 8.6% of all cases, participants reported that the situation emerged during the lockdown. In approximately one third of the cases (34.5%), the maltreatment already started before the lockdown and did not change, in 3.4% of the cases the maltreatment situation improved during the lockdown, and in half of the cases (50.0%) there were concerns before the lockdown and the situation deteriorated during the lockdown. For the remaining 3.4%, participants did not know whether the situations had changed during the lockdown.

### Perpetrators and Risk Factors

In the majority of child maltreatment cases (59.0%) both biological parents were involved as perpetrators of the maltreatment. In 98.3% of the cases, the child was maltreated by at least one of the biological parents, with or without the other biological parent or a stepparent. In the remaining 1.7% of the cases, only a stepparent was involved as perpetrator. In none of the cases, the perpetrator was someone outside the family. In most cases, the biological mother was involved as perpetrator (84.5%), followed by the biological father (63.8%), stepfathers (12.1%), and stepmothers (1.7%).

Risk ratios and 95% confidence intervals of family and child factors are presented in Table 3. Lower parental educational level was the largest risk factor for child maltreatment during the lockdown (RR = 10.28; 95% CI: 3.33–30.74), followed by family size of four or more children (RR = 5.30; 95% CI: 1.27–22.07), and parental unemployment (RR = 3.25; 95% CI: 1.01–10.43). Unemployment was positively related to a family size of four or more children (*r(df)* = .34, *p* = .024). Lower parental education was not related to unemployment (*r(df)* = .24, *p* = .303) or a large family size (*r(df)* = .32, *p* = .157). Immigrant status, single parenthood, stepfamilies, family size of three or more children, age of the child, and gender were not significant risk factors for maltreatment during the lockdown.

**Table 3.** Risk on Child Maltreatment During the Lockdown for Family and Child Factors.

Risk Factors	Risk on Child Maltreatment			
	Exposed, %	Non-exposed, %	Risk Ratio	95% CI
Low educational level	9.8	1.0	10.28^*^	3.33–30.74
Parental unemployment	5.6	1.7	3.25^*^	1.01–10.43
Immigrant (first-generation)	3.4	1.0^a^	3.31	0.49–22.34
Immigrant (second-generation)	1.0	1.0^a^	0.97	0.20–4.72
Single parenthood	3.1	1.9	1.62	0.53–4.98
Stepfamily	7.7	1.9	4.04	0.72–22.60
Family size (3 ≤ children)	4.0	1.7	2.37	0.99–5.65
Family size (4 ≤ children)	9.6	1.8	5.30^*^	1.27–22.07
0–3 years	0.6	1.3	0.47	0.03–6.49
4–11 years	1.6	0.8	1.95	0.99–3.82
12–17 years	0.9	1.3	0.72	0.25–2.03
Gender (girl)	1.2	1.2	1.05	0.51–2.17

*Note*. ^*^Significant risk ratio, indicating an increased risk on child maltreatment of children exposed to the risk factor.

^a^Non-exposed group is the population not belonging to the first-or second-generation immigrants.

## Discussion

Based on reports of professionals we estimated that almost 40,000 children, or 14 per 1,000 children, in the Netherlands were victims of child maltreatment during the first COVID-19 lockdown in the Netherlands (estimate: 39,385; 95% CI: 24,544–54,237; or 8–19 per 1,000 children). This prevalence estimate only applies to children who attend a form of formal childcare or primary or secondary education. Comparison with the prevalence in a similar period without a lockdown for this same population (NPM-2017; Van Berkel et al., 2020) showed that the prevalence of emotional neglect was three times higher during the lockdown. This significant difference was reflected in overall emotional neglect as well as for two main subtypes of emotional neglect: educational neglect and witnessing domestic violence. No significant differences were found for the prevalence estimates of other types of child maltreatment. A striking finding was that only 8.6% of the reported cases the maltreatment emerged during the lockdown. In half of the cases, there were already concerns but these increased during the lockdown to the level of suspected child maltreatment. This implies that the increase in emotional neglect occurred primarily in families with pre-existing problems.

The increase in the number of victims of emotional neglect based on reports of professionals during the lockdown compared to a period without lockdown is consistent with signals of increased contact with hotlines and chat services on child maltreatment and domestic violence during the lockdown (De Kindertelefoon, 2020a, 2020b; Van Bemmel et al., 2020). In addition, it is in line with results of survey-studies and studies based on self-report or parent-report on child maltreatment during a COVID-19 related lockdown (Het Kinderrechten Commissariaat et al., 2020; Rodriguez et al., 2020; Sayfa Çalişmalari, 2020; Vandeviver et al., 2020). However, globally there does not seem to be a consistent increase in official reports of child maltreatment and often even a decline in the number of reports is found (Baron et al., 2020; Đapić et al., 2020; Li & Schwartzapfel, 2020). The difference between self-report and official reports could be related to the under-recognition of emotional neglect and the hidden nature of child maltreatment in general (Glaser, 2002). The fact that there seems to be a decline in official reports while studies based on self-report and reports of professionals point towards an increase in child maltreatment is troubling, since this could indicate that there are more children living in problematic and unsafe situations without being noticed by and receiving help from child welfare professionals (Herrenkohl et al., 2020).

An explanation for the specific increase in emotional neglect during the lockdown could be the disbalance parents experienced between the demands of fulfilling the needs of their children during the lockdown while keeping their own jobs running and their capacity and resources available to meet those demands, since limitations in parents’ capacity to support their children in their emotional and educational needs have been related to an increased risk on emotional neglect, including educational neglect (Van Wert et al., 2018). Another explanation for the increase in emotional neglect could be related to feelings of loneliness parents experienced due to the social-distancing measures. For example, a recent study by Rodriquez et al. (2020) reported that parental feelings of loneliness were related to a 176% increase in the odds of neglecting their children.

The increase in educational neglect shows that not all parents were able to provide proper homeschooling. Although children were provided with schoolwork by their teachers, partially through digital forms of communication, parents needed to support their children in this, especially for children in primary education. Even though this might have been a hard task for most parents, the results of the present study indicate that there were also parents who were not able at all to accomplish this task. In our study, educationally neglecting parents structurally did not support their children with their schoolwork or structurally failed to stimulate their children to participate in their online school activities. A study on home education during the lockdown showed that especially parents with lower educational levels felt less capable to help their children with their schoolwork than higher educated parents (Bol, 2020). The increase in educational neglect, in combination with disproportionally increased struggles in families with low educational levels, is alarming. This might result in growing inequalities regarding educational opportunities.

The increase in the number of children who witnessed domestic violence could be explained by the fact that family members were forced to spend more time together in combination with increased levels of stress. Constantly being together could have led to more tension between and irritations towards each other (Bröer et al., 2021), which could in turn have increased the number of arguments and could eventually have led to domestic violence. Besides the heightened risk for conflicts due to the increased time family members spent together, families also had more arguments due to the reallocation of tasks within the household during the lockdown, as shown in a survey filled out by Italian, British, and American families (Biroli et al., 2020).

Some family factors that were found to be risk factors for maltreatment in earlier research seemed to increase the risk for maltreatment during the lockdown as well. The risk for maltreatment was 10 times larger in families with low educated parents. This finding is in line with the fact that lower educated parents reported they felt less capable in guiding their children with their schoolwork (Bol, 2020), and educational neglect was one of the most prevalent types of maltreatment. This implies that the demands of homeschooling were probably too high for a number of parents, especially for lower educated parents. Lower educated parents often have less recourses to support their children in homeschooling (Bol, 2020) and they are also more often employed in jobs in which working from home was not possible during the lockdown (Yerkes et al., 2020). In unemployed families, the risk for maltreatment was three times larger compared to other families. Family stress may have increased in unemployed families due to financial pressure, and in turn, this stress could have led to more conflicts and harsh parenting (Neppl et al., 2016). Similar to the results of the NPM-2017, the risk for maltreatment was twice as large in families with four or more children compared to smaller families. The demands of homeschooling probably increased with the number of children in the family. Also, living in larger families probably means even less personal space and private time, which might have led to more frustrations and irritabilities between family members, and eventually to the use of more aggressive and abusive behaviors between parents.

Contrary to what was found in previous studies, younger age of the child (0–3 years) did not indicate an elevated risk for child maltreatment during the lockdown compared to older ages (4–17 years). This may be explained by the fact that one of the most prevalent subtypes of maltreatment was educational neglect, which does not apply to this young age. Further, migration background, single parenthood, and stepfamilies were no significant risk factors for maltreatment in the current study. However, considering the relatively small sample size and missing data on some of the risk variables, results on risk factors need to be interpreted with caution.

In addition, the prevalence rates in this study are estimates that have to be interpreted with care. First of all, the prevalence estimates from the two different phases yielded different results. Whereas comparison between the prevalence estimates of both phases combined (or only the prevalence estimate based on the second phase) with prevalence estimate from a period without lockdown suggested maltreatment was increased during the lockdown, the comparison with only the prevalence estimate of the first phase suggested there was no significant difference in maltreatment during the lockdown compared to a period without lockdown. On top of that, as in previous prevalence studies using a similar design (NIS, NPM; Euser et al., 2010; Euser et al., 2013; Sedlak et al., 2010; Van Berkel et al., 2020) we relied on sentinel reports and these were not verified by official agencies. It has to be noted that official proof of maltreatment is incredibly difficult and is often only used in court cases. Nevertheless, there may have been cases where sentinels did not accurately describe the situation, which could have led to an overestimation of the maltreatment prevalence.

Moreover, even though the methods used in the present study were based on the design of the NPM-2017, there were a few differences in the procedure that might have led to differences in results. In contrast to the NPM-2017, during the lockdown contact between children and professionals was limited and most communication took place using digital communication tools. A consequence might have been that signals of maltreatment were harder to notice during the lockdown, which could have resulted in an underestimation of the number of children who were maltreated during this period. In addition, participants from the present study were asked to report about the maltreatment retrospectively, whereas participants reported prospectively in 2017. So, participants in the present study might have been prone to recall bias, even though the period they had to think back on only concerned a couple of months and they reported on children they generally knew well. Their memories might also have been biased because of the media coverage of concerns of child maltreatment during the lockdown, which might have increased awareness, a higher number of reports and therefore an overestimation. However, since all reports of maltreatment were coded by trained coders, including two coders who were also involved in the coding procedure in the NPM-2017, the chance that unfounded concerns were included in the estimate of child maltreatment was minimal. Hence, it seems unlikely that the prevalence estimate during the lockdown is a substantial overestimation. This is supported by the finding that the number of cases in which the professionals indicated to have suspected child maltreatment was almost twice as high as the number of cases of child maltreatment they reported by filling in the online registration form.

In addition, the sample and areas served in the present study and in the NPM-2017 were found to differ on some aspects. The areas served were similar in the percentage of urbanity, but the percentage of households receiving social security benefits in the neighborhoods in which participating organizations were located appeared to be a little lower in the current sample compared to the 2017 sample. This might indicate that the current sample could be considered as less of an at risk group than the sample of the NPM-2017. However, it should be mentioned that the percentage of households receiving social security benefits in the Netherlands also decreased in the general population from 2017 to 2020. Still, the results demonstrate that the higher prevalence estimate of child maltreatment during the lockdown could not be explained by an oversampling of informants from neighborhoods with a lower socioeconomic status in the present study compared to the NPM-2017.

Furthermore, the high non-response rate is a limitation of our study. The high non-response affected the reliability and resulted in larger confidence intervals around the prevalence estimates and risk ratios. The non-response was higher than in the NPM-2017, which is probably because informants were invited by email via directors of organizations this time whereas they were invited by phone by the researchers themselves in the NPM-2017. We expected that this would be the case and therefore invited more organizations to participate in the study. During the first phase of the study there the non-response rate was high in primary and secondary education, whereas in the second phase it was high in childcare organizations. The non-response in the first phase might also have to do with the timing of the study, right after the reopening of the schools and just before the summer break. Childcare setting reopened a little earlier and did not experience pressure to finish the curriculum before the end of the school year, which might have resulted in childcare professionals having more time to participate in this study compared to teachers. An explanation for the non-response of childcare professionals in the second phase of the study might be that at that time they had an increased workload due to high sickness absence due to COVID-19 symptoms and delays in COVID-19 test assessment (Branchevereniging Maatschappelijke Kinderopvang, 2020). Since teachers were granted priority access to COVID-19 tests this was not so much a problem for these occupational branches. Considering that the non-response in the separate phases of our study could be explained by the high workload for teachers in the first phase and for childcare professionals in the second phase of the study, we see the prevalence estimate based on the combination of the two phases as the most complete and reliable estimate of child maltreatment during the lockdown.

Our results indicate that the closure of the schools and childcare settings might have led to unsafe home situations in vulnerable families and greater inequality in educational opportunities. In case the closure of schools and childcare settings is inevitable, regular contact with children and parents must be facilitated. Vulnerable families should be actively approached with extra support to help parents and children with homeschooling, providing a safe environment and ensuring some time and space to escape unsafe home situations. The COVID-19 pandemic globally has had an enormous impact on individuals and society at large. Secondary effects such as on child safety should not be overlooked.
